# Micromechanical Deformation Processes and Failure of PBS Based Composites Containing Ultra-Short Cellulosic Fibers for Injection Molding Applications

**DOI:** 10.3390/polym14214499

**Published:** 2022-10-24

**Authors:** Laura Aliotta, Mattia Gasenge, Vito Gigante, Andrea Lazzeri

**Affiliations:** 1 Department of Civil and Industrial Engineering, University of Pisa, 56122 Pisa, Italy; 2 National Interuniversity Consortium of Materials Science and Technology (INSTM), 50121 Florence, Italy

**Keywords:** polybutylene succinate (PBS), mechanical properties, biocomposites, cellulosic fibers

## Abstract

The use of biobased thermoplastic polymers has gained great attention in the last years as a potential alternative to fossil-based thermoplastic polymers. Biobased polymers in fact offer advantages not only in terms of reduced dependence on fossil resources but they also lower the CO_2_ footprint in accordance with sustainability and climate protection goals. To improve the properties of these materials, reinforcement with biobased fibers is a promising solution; however, it must be kept in mind that the fibers aspect ratio and the interfacial adhesion between the reinforcement and the matrix plays an important role influencing both physical and mechanical properties of the biocomposites. In this paper, the possibility of producing composites by injection molding, based on polybutylene succinate and ultra-short cellulosic fibers has been explored as a potential biobased solution. Thermo-mechanical properties of the composites were investigated, paying particular attention to the local micromechanical deformation processes, investigated by dilatometric tests, and failure mechanisms. Analytical models were also applied to predict the elastic and flexural modulus and the interfacial properties of the biocomposites. Good results were achieved, demonstrating the that this class of biocomposite can be exploited. Compared to pure PBS, the composites with 30 wt.% of cellulose fibers increased the Young’s modulus by 154%, the flexural modulus by 130% and the heat deflection temperature by 9%.

## 1. Introduction

The interest in biobased polymers has grown consistently in recent years thanks to the increased environmental conscientiousness of society, coupled with the fear of the depletion of fossil-based plastics. It is expected that biobased polymers production will grow at a rate of approximately 10% despite up-to-date biobased polymers representing around 10–15% of the entire plastic market [1]. In this context, a significant contribution to the bioplastic market is made by biocomposites, where natural fibers (such as hemp, flax, bamboo, cellulose, sisal etc.) are embedded into the biopolymeric matrix [2,3,4,5,6,7,8,9,10]. The addition of natural fiber is noteworthy; they are renewable, biodegradable, lightweight and less abrasive than tooling [11]. Furthermore, the great variety of natural fibers available has also prompted many researchers to investigate less common natural fibers [12,13,14,15], expanding the biocomposites’ potential. On the other hand, natural fibers offer well-known disadvantages related to moisture uptake and low adhesion with polymeric matrices that leads in many cases to a worsening of the material’s final strength. However, the last aspect can be easily overcome by treating the fibers properly to improve the fiber/matrix adhesion [16,17,18,19,20,21].

Thermoplastic matrices coupled with natural fibers has mainly been exploited for being lightweight, their good insulation properties or for enhancing biodegradability [22,23]. With the development of injection molding technology in the latter half of the 20th century and the deepening of composite materials science, thermoplastic composites have received greater attention thanks to their design flexibility and easy recyclability [24]. For injection molding applications, short fibers are generally exploited due to their low aspect ratio, which makes them easier to process and eliminates concern about the “waviness effect”, which can reduce the load bearing capacity of fibers in longer fiber composites [25,26].

In the 21st century, boosted by serious environmental problems, the coupling of biobased and biodegradable matrices with low-cost natural fibers started to be investigated both in academia and industry [27]. Among the commercial high performance biodegradable polymeric matrices, aliphatic polyesters have high potential; in particular, poly(butylene succinate) (PBS) is considered highly promising as a commercial commodity polymer thanks to its good strength and toughness that is very close to LDPE [28,29]. The variety of natural fibers that can be added to the PBS is very wide; in particular, short wood fibers and their derivatives are widely used as natural reinforcements due to their low cost and high versatility [30]. Ultra-short cellulosic fibers have been investigated in several works in which they have been embedded into a poly(lactic acid) (PLA) matrix. Despite the relatively poor adhesion and the low aspect ratio of the short cellulosic fibers, the composites showed good processability and enhanced stiffness [31,32,33,34].

In this work, it was decided to explore new biocomposites for injection molding applications in which ultra-short cellulosic Arbocel fibers were added, in different amounts, into a PBS matrix. Particular attention was dedicated to the investigation of the mechanical properties, correlating them to micromechanical failure mechanisms; the latter were determined with help of a videoextensometer that was able to measure axial and transversal elongation for calculating the composite volume evolution during tensile tests [35].

Composites are heterogeneous systems in which fibers or fillers can have elastic properties which differ greatly from those of the matrix. The mechanical properties of the composites system are influenced by several factors such as the composition, interfacial interactions, fibers geometry and fibers aspect ratio. The macromechanical response in natural fibers-reinforced composites has been observed to also be influenced by several competitive processes that may occur, depending on the fiber aspect ratio and interfacial adhesion [31]. Recently [36], it has been proved that the mechanical response of ultra-short fiber composites differs from long-fiber composites and the load transferring from the matrix to the fibers occurs not just through shear stresses at the surface along the length of the fibers, but also through normal stresses at the end sections of the fibers (edges or ends effects); in long-fiber composites, the ends effect is negligible, but for short-fibers it is relevant.

Considering that the main feature of composite materials in general is the tailoring of their properties depending on application needs; the study of the properties of new composite materials is markedly relevant from the materials design point of view. Furthermore, given the complexity that characterizes natural materials’ structure and microscopic mechanisms, the testing and modelling of new combinations of natural short fibers with biobased and biodegradable matrices is not trivial, but it is this complexity that makes characterization works much needed. Indeed, to better exploit this category of materials, more knowledge on its microscopic mechanisms and on its design must be developed.

Whereas the dominating micromechanical deformation process is responsible of the final macromechanical performances of the composite, the aim of this work was to thoroughly investigate the micromechanical deformation processes and correlate these results to the macromechanical response. Analytical modelling was also performed to predict the interfacial adhesion and the stiffness properties (flexural and tensile elastic modulus), also evaluating, thanks to the heat deflection temperature (HDT) test, the variations of the thermal resistance of the PBS/cellulose composites analyzed.

## 2. Materials and Methods

### 2.1. Materials

The polybutylene succinate (PBS) used in this work was BIOPBS FZ71PM, purchased from Mitsubishi (Tokyo, Japan); it is a biobased PBS semi-crystalline polyester suitable for injection molding applications. Highly pure ultra-short cellulose microfibers ARBOCEL BE600/30 PU by J Rettenmaier and Sohne (Rosenberg, Germany) were added to the PBS matrix. These fibers had an average mean diameter of 20 µm, a mean fiber length of 60 µm (thus a mean aspect ratio of 3), a bulk density of 200–260 g/L, and a fiber density of 1.44 g/cm^3^). In this work, these fibers will be named Arbocel. The raw material’s relevant properties are summarized in Table 1.

### 2.2. Composites Preparation

PBS based composites, containing different amounts of Arbocel fibers (from 10 up to 30 wt.%) were produced by extrusion compounding according to the compositions reported in Table 2. The extrusion compounding of pure PBS and its composites was carried out using a semi-industrial co-rotating double twin screw extruder Comac EBC 25HT (L/D = 44) (Comac, Cerro Maggiore, Italy); all the materials were dried in a Piovan DP 604–615 dryer (Piovan S.p.A., Venezia, Italy) before the extrusion. The extruder temperature profile from zones 1 to 11 was: 110/135/150/155/155/150/145/135/135/130/120 with the die zone at 120 °C. The extruder flow rate was set at 15 kg/h with a screw speed of 270 rpm. 

### 2.3. Specimens Preparation by Injection Molding

The extruded pellets were sent to Megatech H10/18 injection molding machine (TECNICA DUEBI s.r.l., Fabriano, Italy) for the injection molding of dog-bone specimens (ISO 527-1A, width: 10 mm, thickness: 4 mm, useful length: 80 mm) that are useful for mechanical characterizations.

The nozzle of the injection molding machine was thermo-controlled and squeezed the material inside the mold which was water cooled; after cooling time the specimen was extracted by extractor pins that hit with a precise pressure the specimen to eject it from the open mold. The main injection molding parameters are reported in Table 3.

The temperatures adopted for the injection molding (screw temperature profile and mold temperature) were the same for pure PBS and its composites; however, with the addition of Arbocel fibers and the increasing of their content, the injection and maintenance of the pressure were increased to achieve the correct filling of the mold.

### 2.4. Mechanichal Tests

Mechanical characterizations were performed 3 days after injection molding, keeping the specimens inside a dry keeper (SANPLATEC Corp., Osaka, Japan) at a controlled atmosphere (room temperature and 50% of humidity).

An MTS Criterion model 43 universal testing machine (MTS Systems Corporation, Eden Prairie, MN, USA) was used to perform tensile tests at room temperature. The conditions adopted for the tensile tests were a crosshead speed of 10 mm/min and a load cell of 10 kN interfaced with the MTS Elite Software. At least five specimens were tested for each composition and the average values were reported.

Dilatometry tests were carried out during ensile tests, recording in real time the transversal and axial specimen elongations using a video extensometer (GenieHM1024 Teledyne DALSA camera) connected to the ProVis software (Fundamental Video Extensometer). In this way, during the tensile tests, it was possible to monitor the data in real time and transfer them to the MTS Elite software to measure not only the axial and transversal strains but also the load value. The volume strain was calculated, assuming the two lateral strain components to be equal, according to the Equation (1) [37]:

(1) $$\frac{\Delta V}{V_{0}}=\left(1+\varepsilon_{1}\right){\left(1+\varepsilon_{2}\right)}^{2}-1$$

where the volume variation is Δ*V*, the starting volume is *V*_0_, *ε*_1_ is the axial (or longitudinal) strain and *ε*_2_ is the lateral strain.

The above-mentioned MTS machine, configured in a three-point bending (3PB), was adopted for the flexural tests necessary for the evaluation of the flexural modulus. The flatwise specimen position was used. The crosshead speed was set at 2 mm/min, and the sample dimensions for the 3PB tests were: 80 × 10 × 4 mm. At least five samples for each formulation were tested and the mean flexural modulus value was reported. The ASTM D790 was used for the calculation of flexural modulus (*E_B_*) from stress–strain curves; the equation adopted is reported as follows:

(2) $$E_{B}=\frac{L^{3}m}{4bd^{3}}\;$$

where *L* is the support span; *b* and *d* are the width and the thickness of the sample tested, respectively; and m is the angular coefficient of the linear elastic part of the load–deflection curve (N/mm).

The heat deflection temperature or heat distortion temperature (HDT) is the temperature at which a material begins to deform when a specific load is applied. HDT is essential during the design and production of thermoplastic elements to delimit the maximum temperature at which the material can operate without undergoing to significant deformations under load. The determination of HDT was carried out on a HVT302B (MP Strumenti, Milano, Italy) in accordance with ISO 75-1 (method A). The sample, with a parallelepiped geometry (80 × 10 × 4 mm), was immersed in a silicone oil bath, then was subjected to a flexural stress of 0.45 MPa at the midpoint of the flatwise position of a 3PB configuration. The silicon oil bath was heated with a heating ramp of 120 °C/h; when the sample bar reached a deflection equal to 0.34 mm, the corresponding bath temperature at which this event occurs was the HDT (Type A) value. At least four specimens for each composition were tested and the average value was reported.

### 2.5. Morphological Characterization

Scanning electron microscopy (SEM) was fundamental to confirming the presence and the typology of the micromechanical deformation processes detected by the videoextensometer during tensile tests. The fracture surface of specimens broken during the tensile test offered the best reliable information about the deformation mechanism. Consequently, to study the morphology after the tensile test, the specimens were cryo-fractured along the tensile direction. The new fracture surfaces were investigated by EM-30N scanning electron microscope (SEM) (Coxem Ltd., Daejeon, Korea). The specimens were coated, using a sputter coater Edward S150B, with a thin layer of gold prior to microscopy to avoid charge build up.

### 2.6. Thermal Characterization

The biocomposites; thermal properties were evaluated by differential scanning calorimetry (DSC) using a Q200-TA DSC (TA Instruments, New Castle, DE, USA) equipped with an RSC 90 cooling system. Nitrogen was used as purge gas. A few milligrams (about 15 mg) were cut from the injection molded samples and were subjected to the following heating program: heated at 10 °C/min to 190 °C to delete the thermal history followed by a cooling scan at 20 °C/min up to −50 °C; then a second heating scan from −50 °C to 190 °C, at 10 °C/min, was carried out. The melting temperatures (T_m_) of the PBS and its composites were recorded at the maximum of the melting peak; the melting enthalpy was determined from the corresponding peak area in the thermograms. The PBS crystallinity, *X_c_*, was calculated according to the following Equation (3) [38]:

(3) $$X_{c}=\frac{\Delta H_{m}}{wt.{\%}_{PBS}\Delta H_{m}^{{}^{\circ}}}\cdot 100$$

where $\Delta H_{m}$ is the melting enthalpy of PBS, $\Delta H_{m}^{{}^{\circ}}$ is the melting enthalpy of PBS 100% crystalline and is equal to 110.3 J/g [38]; *wt.*%*_PBS_* is the polymeric mass fraction in the composite.

## 3. Theoretical Analysis

In this work, several analytical models were applied to this class of biocomposites to predict the main mechanical properties. The biocomposites were filled with ultra-short fibers (having an aspect ratio <10) and, for this reason, the choice of the analytical models to be applied required specific considerations. With such a low aspect ratio, these fillers teeter on the boundary between short fibers and particulate fillers class. To corroborate this similarity, as has already been mentioned, in short fiber composites, the transferring of the load from the matrix to the fibers involves not only the cylindrical surfaces of the fibers but also the fibers’ ends [36,39].

### 3.1. Elastic Modulus

The composite system analyzed in this study was characterized by a thermoplastic matrix in which ultra-short fibers, randomly oriented, were embedded. The prediction of the elastic modulus is not trivial, and much information correlated to geometrical, topological and mechanical parameters is necessary [40]. When a theoretical approach is followed, it is necessary to collect as much information as is available (such as the mechanical properties of the matrix and the fibers, the fibers volume fraction, etc.) and then to cover the missing data with suitable assumptions. In this context, for the prediction of the elastic modulus, the existing analytical expressions available were obtained either from the elasticity theory or from the attempt to fit experimental data with theoretical curves by adding suitable constants to existing theoretical models [39,41,42,43,44]. The analytical models adopted in this work are reported in Table 4.

In Table 4, *E_f_* and *E_m_* are the elastic modulus of the fibers and matrix, respectively, *ϕ_f_* is the fiber volume fraction, *a_r_* is the fibers’ aspect ratio, υ is the Poisson ratio of the matrix (≈0.35), and *P* is the fibers’ packing factor with the value 2π/√3. In the Halpin–Tsai model, the two terms $E_{l}$ and $E_{t}$ are, respectively, the longitudinal and the tangential modulus, which can be calculated as follows:

(4) $$E_{l}=E_{m}\cdot \frac{1+2\cdot a_{r}\cdot \left(\frac{\frac{E_{f}}{E_{m}}\;-\;1}{\frac{E_{f}}{E_{m}}\;+\;2a_{r}}\right)\cdot \phi_{f}}{1-\left(\frac{\frac{E_{f}}{E_{m}}\;-\;1}{\frac{E_{f}}{E_{m}}\;+\;2a_{r}}\right)\cdot \phi_{f}}\;$$

(5) $$E_{t}=E_{m}\cdot \frac{1+2\cdot a_{r}\cdot \left(\frac{\frac{E_{f}}{E_{m}}\;-\;1}{\frac{E_{f}}{E_{m}}+2}\right)\cdot \phi_{f}}{1\;-\;\left(\frac{\frac{E_{f}}{E_{m}}\;-\;1}{\frac{E_{f}}{E_{m}}\;+\;2}\right)\cdot \phi_{f}}\;$$

To compute the model listed in Table 4, apart from the data taken from experimental measurements, some initial data or assumptions must be taken and are listed in Table 5.

### 3.2. Interfacial Adhesion

Interfacial adhesion between the reinforcement and the matrix is fundamental because it influences both the physical and mechanical properties of the composites. Nevertheless, its experimental determination is often laborious. For this reason, analytical models are frequently adopted to calculate the interfacial shear stress (IFSS) using simple mechanical properties that can be obtained from the stress–strain curves of the matrix and of the composites.

In this context, the Pukánszky model has been largely adopted with success in particulate filled and short fiber composites systems [31,33,49]. The model considers that the composite strength varies with the fiber load according to the following equation:

(6) $$\sigma_{c}=\sigma_{m}\frac{1-V_{f}}{1+2.5V_{f}}\exp\left(BV_{f}\right)$$

where *σ_c_* and *σ_m_* are the strength at break of the composite and matrix, respectively, *V_f_* is the volume fiber fraction. The term (1 − *V_f_*)/(1 *+* 2.5*V_f_*) is correlated to the reduction of the effective load-bearing cross section caused by the addition of the fibers; *B* is an interaction parameter connected to the efficiency of stress transmission from the matrix to the fiber. Linearizing Equation (6) in Equation (7), it can be observed that the linear correlation can be obtained in which the interaction parameter, *B*, can be calculated as the slope of the Pukánszky’s plot (obtained plotting the natural logarithm of Pukánszky’s reduced strength, *σ_red_*, against the volume fraction of the reinforcement).

(7) $$\ln\sigma_{red}=\ln\frac{\sigma_{c}\left(1+2.5V_{f}\right)}{\sigma_{m}\left(1-V_{f}\right)}=BV_{f}$$

## 4. Results and Discussion

**Figure 1:**
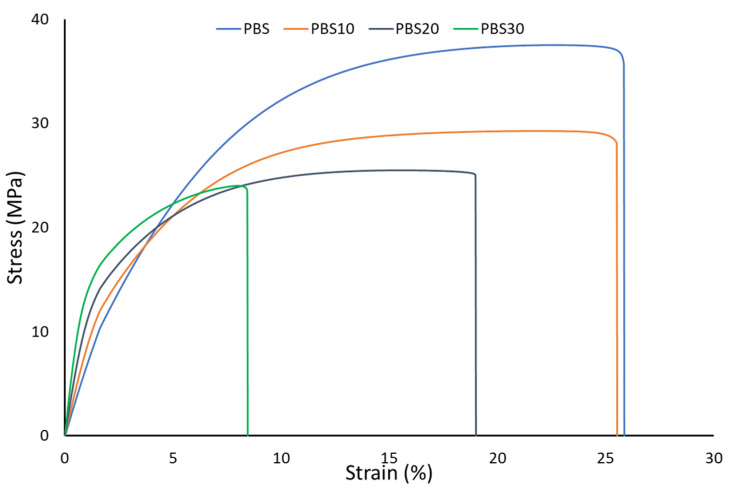
Stress–strain curves of PBS and its composites.

**Figure 2:**
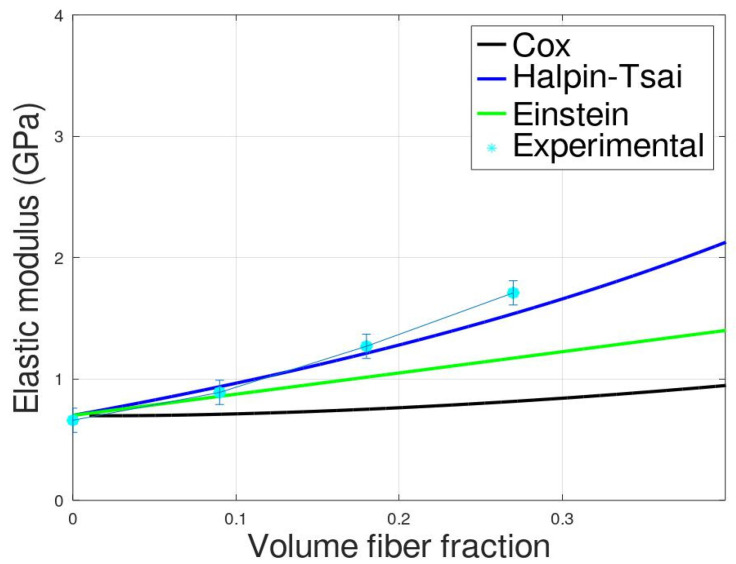
Comparison between experimental elastic modulus of the composites and the analytical models.

In Figure 1 are shown the representative stress–strain curves obtained from mechanical tests. Increasing the fiber content increases the elastic modulus (observable in an increase in the slope of the elastic part of the stress–strain curve); this trend agrees with other literature studies in which cellulosic fibers were used [33,34,50]. The Young’s modulus increment is ascribed to the higher stiffness of the fibers that increments the polymeric matrix stiffness. The results of the prediction of the Elastic modulus compared with the experimental data, reported in Figure 2, show that the Cox model provides underestimated stiffness prediction.

This discrepancy between Cox prediction and the experimental data can be ascribed to the fact that the Cox model derives from the shear lag theory in which the fibers are long and aligned unidirectionally; furthermore, the composite behavior as well as the matrix behavior are considered rigid and perfectly elastic [41]. The fibers used for this work were ultra-short, randomly oriented and from the stress–strain curves it is evident that the mechanical behavior is elasto-plastic. The latter issue can be responsible for the main discrepancy observed between the predicted value and the experimental data; in fact, despite the Cox model being originally used for predicting long fiber composites systems, good predictions were also found with short fiber composites [25,51,52]. Another aspect for which the Cox model fails is correlated with the ultra-short aspect ratio of the fibers (<10) that, as recently demonstrated [36], makes it impossible to neglect the stress at the fiber ends.

Although the Einstein model is valid for particulate composites, the very low aspect ratio of the fibers makes it worth noting to be validated. In fact, it has been observed in the literature [53,54,55] that reliable results were achieved when the Einstein model was applied to short fiber composites systems. For the PBS/Arbocel composites, the Einstein approaches slightly improve the fitting with experimental data; however, the data fitted are still underestimated. When the fiber content is low (PBS10), the fitting is very good and the major discrepancies are observed, increasing the fiber content in agreement with what was observed in the literature for other short fiber composites systems [55].

The Halpin–Tsai model fits the experimental data quite well; this model, in fact, is largely adopted for the prediction of composites with unaligned short fibers; this model, containing the expression for the evaluation of both transversal and longitudinal modulus, provides an acceptable fitting.

**Figure 3:**
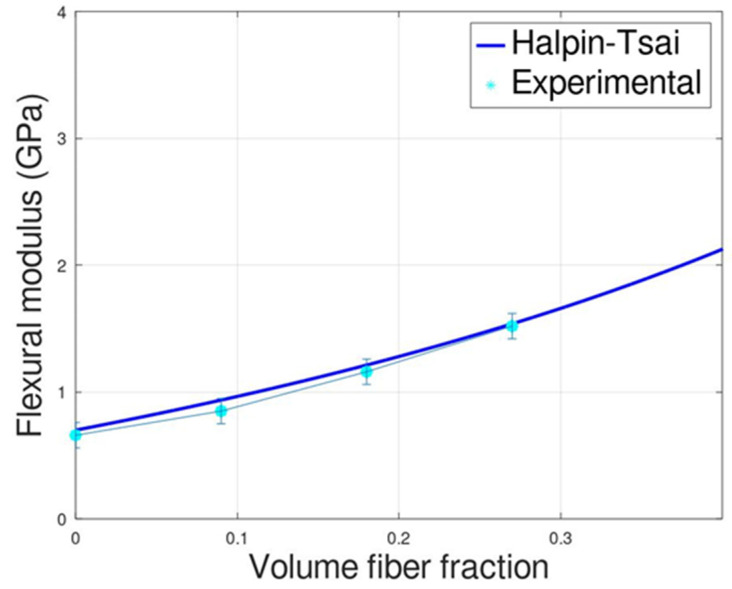
Comparison between experimental flexural modulus of the composites and the analytical models.

Concerning the flexural properties, the results obtained are coherent and in line with the elastic modulus, with an increment of the flexural modulus with the fiber content. Consequently, based on the results of the elastic modulus analytical modeling, the Halpin–Tsai model was applied to the flexural modulus. The analytical fitting compared with experimental data (reported in Figure 3) shows a very good correlation demonstrating that the Halpin–Tsai model can be used for the prediction of both elastic and flexural modulus.

**Figure 4:**
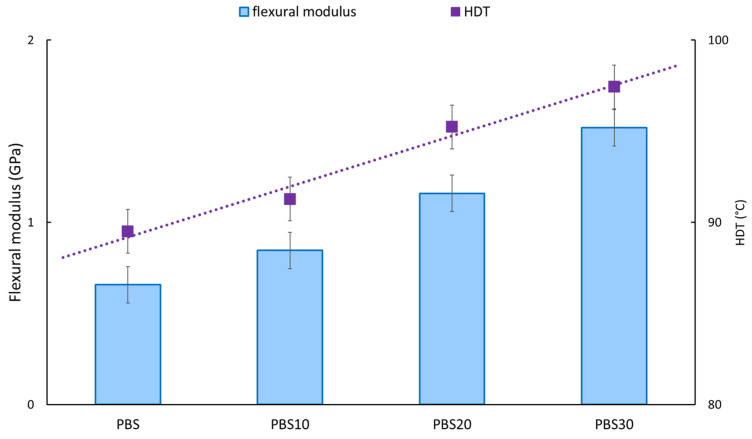
Flexural modulus and HDT results.

The increment of the flexural modulus is also strictly connected to the growth of HDT, as observed and reported in Figure 4. These results could be expected as the HDT test is a 3PBD test in temperature sweeping [56]. The HDT test provides important information for the composite design because it is a measure of the upper boundary of the dimensional stability and strength of the material under a fixed load and a particular range of temperature conditions. HDT measures the ability of the material to retain its stiffness at elevated temperatures. The increment of the composite stiffness with the fiber content indicates a reduction in the free volume present in the system, which enhances the dimensional stability of the composites and, hence, the HDT values [57].

The fibers, having a higher thermal stability and flexural modulus with respect to the pure PBS matrix, lead to an increment of the flexural properties and, at the same time, to the thermal stability of the injection molded materials.

From the main thermal properties of pure PBS and its composites, summarized in Table 6, no significant differences in the melting temperature, glass transition temperature and crystallinity degree of the neat polymer and its composites can be observed in agreement with what was observed in other similar systems [58,59,60].

**Figure 5:**
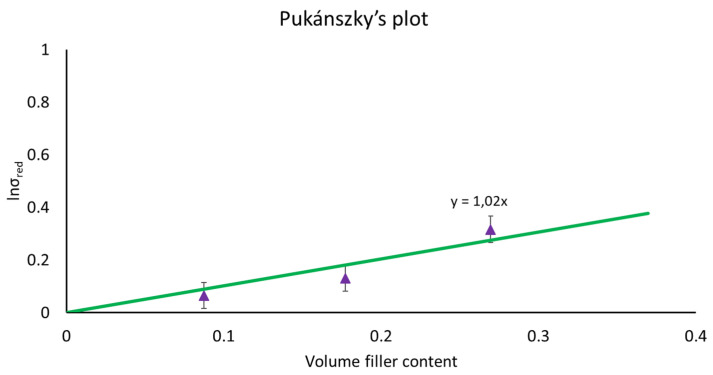
Pukánszky’s plot for PBS composites with Arbocel.

Regarding the tensile properties, it can be observed that the addition of 10 wt.% of Arbocel does not significantly change the elongation at the break of the composites while the stress at break decreases. On increasing the Arbocel content, a progressive reduction of elongation at break and stress at break was observed. The mechanical results suggest that a slight or entirely null stress transfer takes places between the fiber and the matrix; consequently, the fibers only reduce the load bearing section leading to a decrement of the stress at the break. The poor adhesion between the fibers and the matrix was also confirmed by the B parameter obtained from the Pukánszky’s plot, reported in Figure 5, in which a low B value equal to 1.02 was obtained.

**Figure 6:**
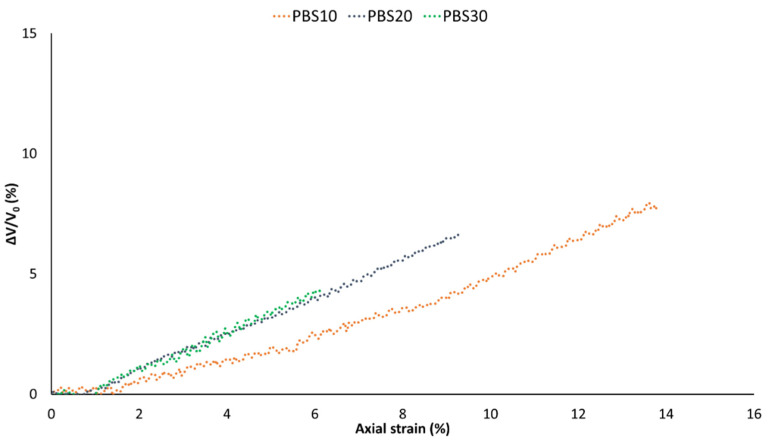
Volume strain-strain curves for PBS composites.

Several local processes (shear yielding of the matrix, cavitation, debonding etc.) can occur when the composite undergoes tensile deformation; these mechanisms cause volume variation and may occur simultaneously or consecutively [49]. The interface adhesion plays a fundamental role; in fact, changes in adhesion can considerably modify the dominating deformation process and consequently the failure mechanism [31]. The dilatometric results reported in Figure 6 allow us to correlate the mechanical properties obtained with the failure mechanisms. Some local events (like fibers debonding or fibers pull-out) cause an apparent volume variation (volume occupied by the specimen, which is different from the volume occupied effectively by the material because of the porosity and voids).

The dilatational response of the composites analyzed, when they are subjected to an applied stress, lead to an appreciable deformation in the material bulk that causes volume variations detectable with the use of the videoextensometer and calculated according to Equation (1).

In Figure 6, a significant volume variation, individuated by a significant change in the slope of the volume change as a function of the axial elongation, can be observed. The point at which the slope change occurs is followed by an abrupt increment of the volume variation. Considering the low adhesion between the fibers and the matrix (detected by mechanical results and the low B parameter), the related process must be the fibers debonding. Generally, debonding is the dominating process that takes place during deformation when low fibers/matrix adhesion is present and in the absence of a coupling agent [49,61]. Consequently, from the intersection point at which the slopes change occurs, it is possible to calculate the axial elongation at which debonding appears and, from the stress–strain curves, it is possible to calculate the stress at debonding. For the composites system analyzed, the debonding stress is almost the same for all fiber amounts; nevertheless, a slight increment of the stress at debonding with the fiber content was registered [37,62]. From PBS10 to PSA30, the stress at debonding registered was: 10.91 MPa, 11.12 MPa and 12.67 MPa. According to the literature, the fibers with the largest diameters debond first; it is not the aspect ratio or average size, but the size and the number of thick fibers determine the initiation of the debonding process [31]. Increasing the fibers amount, the probability of finding a high number of thinner fibers rise and the stress at the debonding increase. However, increasing the fiber amount also increases the number of fibers that debond and thus generate voids around the fibers; this leads to an increment of the volume slope variation as confirmed by the dilatometric curves in which by increasing the fibers quantity, the volume slope variation increases.

**Figure 7:**
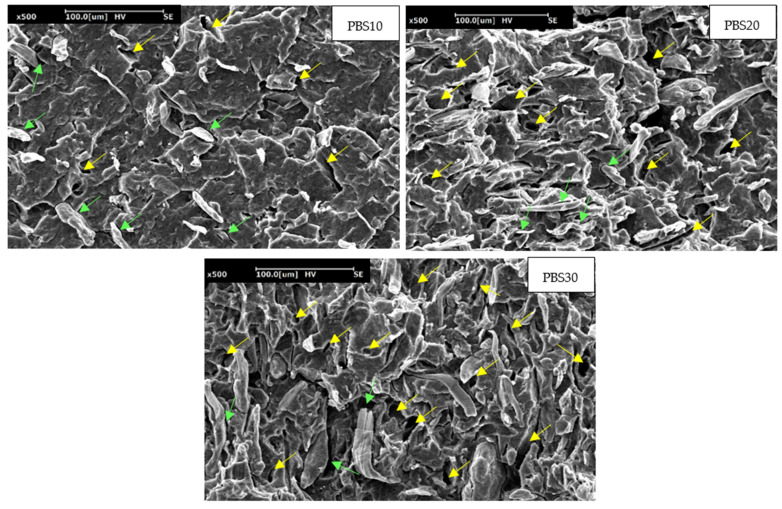
SEM micrographs made at the surface of tensile specimens cryo-fractured along the draw direction for PBS10, PBS20 and PBS30.

The SEM micrographs at the cryo-fractured surface of the tensile specimens along the draw direction (Figure 7) support the dilatometric results.

It can be observed that, effectively, voids are present around the fibers (green arrows) caused by the low fiber/matrix adhesion that is the response to the fibers debonding. Increasing the fiber quantity, the debonded fibers that generated voids are present in a large number at the interface, leading to a volume change increment. Nevertheless, debonding is the dominating deformation mechanism but, due to the poor adhesion, the fiber debonding is accompanied by fiber pull-out that leaves “holes” clearly visible in the micrographs (yellow arrows) and that are also responsible for the volume strain increment. It can be observed that, by increasing the fiber quantity, the fibers pull-out is higher with an increasing number of holes generated. The higher stress at debonding generates higher pull-out fibers. The results obtained are in agreement with similar studies, in which, in fibers with poor adhesion and low aspect ratio, debonding was proved to be the main micromechanical deformation mechanism, often accompanied by fibers pull-out (that can occur simultaneously) [31,49,63,64].

## 5. Conclusions

In this work, the properties of PBS composites reinforced with commercial cellulosic fibers (Arbocel) with a very low aspect ratio were investigated. Summarizing the results, two main aspects were deepened: the fiber–matrix interface adhesion and the stiffness depend on the fiber volume fraction. The composites’ tensile properties are strongly interconnected with the fiber/matrix adhesion. In this context, a low adhesion was registered that led to a decrement of the tensile strength and elongation at break. The micromechanical deformation process, determined by dilatometric tests coupled with SEM analysis, revealed that there is a close correlation between the composite strength and micromechanical deformation processes. Debonding, often coupled with fibers pull-out, was responsible for the tensile strength decrement and volume strain increment.

The stiffness of the composites was predicted by applying and comparing different analytical models. It was found that the Halpin–Tsai model gives a good fitting not only for the tensile elastic modulus but also for the flexural modulus. The increment of the flexural modulus was related to the HDT increment with the growth of the fiber content.

Based on the results obtained, it emerged that Arbocel fibers, due to their very low aspect ratio, show a very good processability and can be easily adopted for the injection molding application, providing a fully biobased solution for those applications where high stiffness and thermal resistance are required. However, the poor fiber/matrix adhesion does not allow us to efficiently improve the tensile strength of the composites; consequently, further work must be carried out to enhance the fiber–matrix adhesion to enlarge the potential application field of these biocomposites.

## Figures and Tables

**Table 1:** Raw relevant material properties.

**BIOPBS FZ71PM**	Density: 1.26 g/cm^3^ MFR (190 °C, 2.16 kg): 22 g/10 min
**Arbocel BE600/30 PU**	Average mean diameter: 20 µm Mean fiber length: 60 µm Mean aspect ratio: 3 Bulk density: 200–260 g/L Fiber density: 1.44 g/cm^3^

**Table 2:** Compositions.

Name	PBS wt.%	Arbocel wt.%
PBS	100	-
PBS10	90	10
PBS20	80	20
PBS30	70	30

**Table 3:** Main injection molding parameters.

			PBS	PBS10	PBS20	PBS30
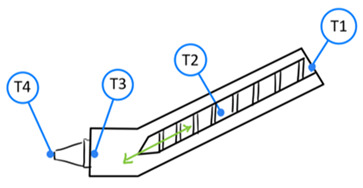	TEMPERATURES (°C)	T1	140	140	140	140
		T2	150	150	150	150
		T3	140	140	140	140
		T4	140	140	140	140
		Mold	50	50	50	50
	INJECTION	Injection pressure (bar)	55	65	75	75
		Injection filling speed (%)	80	80	80	80
	MAINTAINING	Maintaining pressure (bar)	50	60	70	70
		Maintaining time (s)	10	10	10	10
		Cooling time (s)	15	15	15	15

**Table 4:** Summary of the analytical equations adopted in this work for the composites’ Young’s modulus prediction.

Model	E_composite_	Parameters Used
Einstein	$E_{c}=E_{m}\left(1+2,5\;\phi_{f}\right)$	
Cox	$E_{c}=\phi_{m}E_{m}+\phi_{f}E_{f}\cdot \left(1-\frac{\tanh\;\left(n\cdot a_{r}\right)}{\left(n\cdot a_{r}\right)}\right)$	$n=\sqrt{\frac{2E_{m}}{E_{f}\left(1\;+\;\upsilon \right)\;ln\;(\frac{P}{\phi_{f}})}}$
Halpin-Tsai	$E_{c}=\frac{3}{8}E_{l}+\frac{5}{8}E_{t}$	

**Table 5:** Initial modeling data assumption.

Name	Symbol	Value	Source
Fiber modulus	*E_m_*	23.4 (GPa)	[45]
Fiber aspect ratio	*a_r_*	3	From Technical data sheet
Matrix Poisson ration	*υ*	0.35	[46,47,48]

**Table 6:** HDT, glass transition temperature and.

Name	T_g_ (°C)	T_m_ (°C)	ΔH_m_ (J/g)	X_c_ (%)
**PBS**	−34.0	115.1	57.5	52.1
**PBS10**	−34.1	115.1	52.4	52.8
**PBS20**	−34.1	116.0	45.7	51.8
**PBS30**	−34.0	116.7	40.5	52.4

## Data Availability

Not applicable.

## References

[1] Abrha H., Cabrera J., Dai Y., Irfan M., Toma A., Jiao S., Liu X. (2022). Bio-Based Plastics Production, Impact and End of Life: A Literature Review and Content Analysis. Sustainability.

[2] Gupta A., Chudasama B., Chang B.P., Mekonnen T. (2021). Robust and Sustainable PBAT—Hemp Residue Biocomposites: Reactive Extrusion Compatibilization and Fabrication. Compos. Sci. Technol..

[3] Momeni S., Safder M., Khondoker M.A.H., Elias A.L. (2021). Valorization of Hemp Hurds as Bio-Sourced Additives in Pla-Based Biocomposites. Polymers.

[4] Sergi C., Sbardella F., Lilli M., Tirillò J., Calzolari A., Sarasini F. (2020). Hybrid Cellulose–Basalt Polypropylene Composites with Enhanced Compatibility: The Role of Coupling Agent. Molecules.

[5] Wang Q., Ji C., Sun J., Zhu Q., Liu J. (2020). Structure and Properties of Polylactic Acid Biocomposite Films Reinforced with Cellulose Nanofibrils. Molecules.

[6] Liang Z., Wu H., Liu R., Wu C. (2021). Preparation of Long Sisal Fiber-Reinforced Polylactic Acid Biocomposites with Highly Improved Mechanical Performance. Polymers.

[7] Zuccarello B., Marannano G. (2018). Random Short Sisal Fiber Biocomposites: Optimal Manufacturing Process and Reliable Theoretical Models. Mater. Des..

[8] Sánchez M.L., Patiño W., Cárdenas J. (2020). Physical-Mechanical Properties of Bamboo Fibers-Reinforced Biocomposites: Influence of Surface Treatment of Fibers. J. Build. Eng..

[9] Wu Y., Fei M., Chen T., Qiu R., Liu W. (2020). Biocomposites from Bamboo Fibers and Palm Oil-Based Thermosets: Effects of Natural Phenolic Cross-Linkers. Compos. Commun..

[10] Pivsa-Art S., Pivsa-Art W. (2021). Eco-Friendly Bamboo Fiber-Reinforced Poly(Butylene Succinate) Biocomposites. Polym. Compos..

[11] Bos H.L., Müssig J., van den Oever M.J.A. (2006). Mechanical Properties of Short-Flax-Fibre Reinforced Compounds. Compos. Part A Appl. Sci. Manuf..

[12] Botta L., Fiore V., Scalici T., Valenza A., Scaffaro R. (2015). New Polylactic Acid Composites Reinforced with Artichoke Fibers. Materials.

[13] De Rosa I.M., Kenny J.M., Puglia D., Santulli C., Sarasini F. (2010). Morphological, Thermal and Mechanical Characterization of Okra (*Abelmoschus esculentus*) Fibres as Potential Reinforcement in Polymer Composites. Compos. Sci. Technol..

[14] Sarikanat M., Seki Y., Sever K., Durmuşkahya C. (2014). Determination of Properties of *Althaea officinalis* L. (Marshmallow) Fibres as a Potential Plant Fibre in Polymeric Composite Materials. Compos. B Eng..

[15] Komal U.K., Lila M.K., Singh I. (2020). PLA/Banana Fiber Based Sustainable Biocomposites: A Manufacturing Perspective. Compos. B Eng..

[16] Oksman K., Wallström L., Berglund L.A., Toledo Filho R.D. (2002). Morphology and Mechanical Properties of Unidirectional Sisal-Epoxy Composites. J. Appl. Polym. Sci..

[17] Oksman K., Skrifvars M., Selin J.F. (2003). Natural Fibres as Reinforcement in Polylactic Acid (PLA) Composites. Compos. Sci. Technol..

[18] Valadez-Gonzalez A., Cervantes-Uc J.M., Olayo R., Herrera-Franco P.J. (1999). Effect of Fiber Surface Treatment on the Fiber-Matrix Bond Strength of Natural Fiber Reinforced Composites. Compos. B Eng..

[19] Huda M.S., Drzal L.T., Mohanty A.K., Misra M. (2007). The Effect of Silane Treated-and Untreated-Talc on the Mechanical and Physico-Mechanical Properties of Poly (Lactic Acid)/Newspaper Fibers/Talc Hybrid Composites. Compos. B Eng..

[20] Cho D., Seo J.M., Lee H.S., Cho C.W., Han S.O., Park W.H. (2007). Property Improvement of Natural Fiber-Reinforced Green Composites by Water Treatment. Adv. Compos. Mater..

[21] Fiore V., Scalici T., Nicoletti F., Vitale G., Prestipino M., Valenza A. (2016). A New Eco-Friendly Chemical Treatment of Natural Fibres: Effect of Sodium Bicarbonate on Properties of Sisal Fibre and Its Epoxy Composites. Compos. B Eng..

[22] Bledzki A.K., Jaszkiewicz A., Urbaniak M., Stankowska-Walczak D. (2012). Biocomposites in the Past and in the Future. Fibres Text. East. Eur..

[23] Morreale M., Mistretta M.C., Fiore V. (2017). Creep Behavior of Poly(Lactic Acid) Based Biocomposites. Materials.

[24] Manu T., Nazmi A.R., Shahri B., Emerson N., Huber T. (2022). Biocomposites: A Review of Materials and Perception. Mater. Today Commun..

[25] Gigante V., Aliotta L., Phuong V.T., Coltelli M.B., Cinelli P., Lazzeri A. (2017). Effects of Waviness on Fiber-Length Distribution and Interfacial Shear Strength of Natural Fibers Reinforced Composites. Compos. Sci. Technol..

[26] Thirumalai R., Prabhakaran D., Lilholt H., Aviles F., Løgstrup Andersen T., Knudsen H. (2013). Fibre Waviness and Misalignment Measurement of Unidirectional Glass/LPET Commingled Composites—Effect on Mechanical Properties. Risoe International Symposium on Materials Science. Proceedings.

[27] Mohanty A.K., Misra M., Hinrichsen G. (2000). Biofibres, Biodegradable Polymers and Biocomposites: An Overview. Macromol. Mater. Eng..

[28] Shih Y.F., Chen L.S., Jeng R.J. (2008). Preparation and Properties of Biodegradable PBS/Multi-Walled Carbon Nanotube Nanocomposites. Polym. (Guildf.).

[29] Rafiqah S.A., Khalina A., Harmaen A.S., Tawakkal I.A., Zaman K., Asim M., Nurrazi M.N., Lee C.H. (2021). A Review on Properties and Application of Bio-Based Poly(Butylene Succinate). Polymers.

[30] La Mantia F.P., Morreale M. (2011). Green Composites: A Brief Review. Compos. Part A Appl. Sci. Manuf..

[31] Renner K., Kenyó C., Móczó J., Pukánszky B. (2010). Micromechanical Deformation Processes in PP/Wood Composites: Particle Characteristics, Adhesion, Mechanisms. Compos. Part A Appl. Sci. Manuf..

[32] Kain S., Ecker J.V., Haider A., Musso M., Petutschnigg A. (2020). Effects of the Infill Pattern on Mechanical Properties of Fused Layer Modeling (FLM) 3D Printed Wood/Polylactic Acid (PLA) Composites. Eur. J. Wood Wood Prod..

[33] Aliotta L., Gigante V., Cinelli P., Coltelli M.-B., Lazzeri A. (2020). Effect of a Bio-Based Dispersing Aid (Einar^®^ 101) on PLA-Arbocel^®^ Biocomposites: Evaluation of the Interfacial Shear Stress on the Final Mechanical Properties. Biomolecules.

[34] Aliotta L., Gigante V., Coltelli M.B., Cinelli P., Lazzeri A. (2019). Evaluation of Mechanical and Interfacial Properties of Bio-Composites Based on Poly (Lactic Acid) with Natural Cellulose Fibers. Int. J. Mol. Sci..

[35] Aliotta L., Gigante V., Coltelli M.-B., Lazzeri A. (2021). Volume Change during Creep and Micromechanical Deformation Processes in PLA–PBSA Binary Blends. Polymers.

[36] Aliotta L., Lazzeri A. (2020). A Proposal to Modify the Kelly-Tyson Equation to Calculate the Interfacial Shear Strength (IFSS) of Composites with Low Aspect Ratio Fibers. Compos. Sci. Technol..

[37] Aliotta L., Cinelli P., Beatrice Coltelli M., Lazzeri A. (2018). Rigid Filler Toughening in PLA-Calcium Carbonate Composites: Effect of Particle Surface Treatment and Matrix Plasticization. Eur. Polym. J..

[38] Abderrahim B., Abderrahman E., Mohamed A., Fatima T., Abdesselam T., Krim O. (2015). Kinetic Thermal Degradation of Cellulose, Polybutylene Succinate and a Green Composite: Comparative Study. World J. Environ. Eng..

[39] Kim H.G., Kwac L.K. (2009). Evaluation of Elastic Modulus for Unidirectionally Aligned Short Fiber Composites. J. Mech. Sci. Technol..

[40] Bourkas G., Prassianakis I., Kytopoulos V., Sideridis E., Younis C. (2010). Estimation of Elastic Moduli of Particulate Composites by New Models and Comparison with Moduli Measured by Tension, Dynamic, and Ultrasonic Tests. Adv. Mater. Sci. Eng..

[41] Cox H.L. (1952). The Elasticity and Strength of Paper and Other Fibrous Materials. Br. J. Appl. Phys..

[42] Ahmed S., Jones F.R. (1990). A Review of Particulate Reinforcing Theories for Polymer Composites. J. Mater. Sci..

[43] Halpin J.C., Kardos J.L. (1976). The Halpin-Tsai Equations: A Review. Polym. Eng. Sci..

[44] Fu S.Y., Feng X.Q., Lauke B., Mai Y.W. (2008). Effects of Particle Size, Particle/Matrix Interface Adhesion and Particle Loading on Mechanical Properties of Particulate-Polymer Composites. Compos. B Eng..

[45] Adusumali R.B., Reifferscheid M., Weber H., Roeder T., Sixta H., Gindl W. (2006). Mechanical Properties of Regenerated Cellulose Fibres for Composites. Macromol. Symp..

[46] Luo J.J., Daniel I.M. (2003). Characterization and Modeling of Mechanical Behavior of Polymer/Clay Nanocomposites. Compos. Sci. Technol..

[47] Huang Y., Kinloch A.J. (1992). Modelling of the Toughening Mechanisms in Rubber-Modified Epoxy Polymers—Part I Finite Element Analysis Studies. J. Mater. Sci..

[48] Qi D., Hinkley J., He G. (2005). Molecular Dynamics Simulation of Thermal and Mechanical Properties of Polyimide-Carbon-Nanotube Composites. Model Simul. Mat. Sci. Eng..

[49] Bartos A., Kócs J., Anggono J., Móczó J., Pukánszky B. (2021). Effect of Fiber Attrition, Particle Characteristics and Interfacial Adhesion on the Properties of PP/Sugarcane Bagasse Fiber Composites. Polym. Test..

[50] Facca A.G., Kortschot M.T., Yan N. (2006). Predicting the Elastic Modulus of Natural Fibre Reinforced Thermoplastics. Compos. Part A Appl. Sci. Manuf..

[51] Shibata S., Cao Y., Fukumoto I. (2005). Press Forming of Short Natural Fiber-Reinforced Biodegradable Resin: Effects of Fiber Volume and Length on Flexural Properties. Polym. Test..

[52] Serrano A., Espinach F.X., Tresserras J., del Rey R., Pellicer N., Mutje P. (2014). Macro and Micromechanics Analysis of Short Fiber Composites Stiffness: The Case of Old Newspaper Fibers-Polypropylene Composites. Mater. Des..

[53] Aliotta L., Gigante V., Coltelli M., Cinelli P., Lazzeri A., Seggiani M. (2019). Thermo-Mechanical Properties of PLA/Short Flax Fiber Biocomposites. Appl. Sci..

[54] George G., Tomlal Jose E., Åkesson D., Skrifvars M., Nagarajan E.R., Joseph K. (2012). Viscoelastic Behaviour of Novel Commingled Biocomposites Based on Polypropylene/Jute Yarns. Compos. Part A Appl. Sci. Manuf..

[55] Abhilash R.M., Venkatesh G.S., Chauhan S.S. (2021). Micromechanical Modeling of Bamboo Short Fiber Reinforced Polypropylene Composites. Multiscale Multidiscip. Model. Exp. Des..

[56] Aliotta L., Sciara L.M., Cinelli P., Canesi I., Lazzeri A. (2022). Improvement of the PLA Crystallinity and Heat Distortion Temperature Optimizing the Content of Nucleating Agents and the Injection Molding Cycle Time. Polymers.

[57] Panthapulakkal S., Sain M. (2007). Injection-Molded Short Hemp Fiber/Glass Fiber-Reinforced Polypropylene Hybrid Composites—Mechanical, Water Absorption and Thermal Properties. J. Appl. Polym. Sci..

[58] Picard M.C., Rodriguez-Uribe A., Thimmanagari M., Misra M., Mohanty A.K. (2020). Sustainable Biocomposites from Poly(Butylene Succinate) and Apple Pomace: A Study on Compatibilization Performance. Waste Biomass Valorization.

[59] Nanda M.R., Misra M., Mohanty A.K. (2013). Performance Evaluation of Biofibers and Their Hybrids as Reinforcements in Bioplastic Composites. Macromol. Mater. Eng..

[60] Lee S.-H., Wang S. (2006). Biodegradable Polymers/Bamboo Fiber Biocomposite with Bio-Based Coupling Agent. Compos. Part A Appl. Sci. Manuf..

[61] Faludi G., Dora G., Imre B., Renner K., Móczó J., Pukánszky B. (2014). PLA/Lignocellulosic Fiber Composites: Particle Characteristics, Interfacial Adhesion, and Failure Mechanism. J. Appl. Polym. Sci..

[62] Lazzeri A., Thio Y.S., Cohen R.E. (2004). Volume Strain Measurements on CACO3/Polypropylene Particulate Composites: The Effect of Particle Size. J. Appl. Polym. Sci..

[63] Faludi G., Link Z., Renner K., Móczó J., Pukánszky B. (2014). Factors Determining the Performance of Thermoplastic Polymer/Wood Composites; the Limiting Role of Fiber Fracture. Mater. Des..

[64] Várdai R., Lummerstorfer T., Pretschuh C., Jerabek M., Gahleitner M., Faludi G., Móczó J., Pukánszky B. (2020). Reinforcement of PP with Polymer Fibers: Effect of Matrix Characteristics, Fiber Type and Interfacial Adhesion. Polym. (Guildf.).

